# Optical Panel Inspection Using Explicit Band Gaussian Filtering Methods in Discrete Cosine Domain

**DOI:** 10.3390/s23031737

**Published:** 2023-02-03

**Authors:** Hong-Dar Lin, Huan-Hua Tsai, Chou-Hsien Lin, Hung-Tso Chang

**Affiliations:** 1 Department of Industrial Engineering and Management, Chaoyang University of Technology, Taichung 413310, Taiwan; 2 Department of Civil, Architectural, and Environmental Engineering, The University of Texas at Austin, Austin, TX 78712-0273, USA

**Keywords:** touch panel, defect detection, directional texture, discrete cosine transform, frequency domain filtering

## Abstract

Capacitive touch panels (CTPs) have the merits of being waterproof, antifouling, scratch resistant, and capable of rapid response, making them more popular in various touch electronic products. However, the CTP has a multilayer structure, and the background is a directional texture. The inspection work is more difficult when the defect area is small and occurs in the textured background. This study focused mainly on the automated defect inspection of CTPs with structural texture on the surface, using the spectral attributes of the discrete cosine transform (DCT) with the proposed three-way double-band Gaussian filtering (3W-DBGF) method. With consideration to the bandwidth and angle of the high-energy region combined with the characteristics of band filtering, threshold filtering, and Gaussian distribution filtering, the frequency values with higher energy are removed, and after reversal to the spatial space, the textured background can be weakened and the defects enhanced. Finally, we use simple statistics to set binarization threshold limits that can accurately separate defects from the background. The detection outcomes showed that the flaw detection rate of the DCT-based 3W-DBGF approach was 94.21%, the false-positive rate of the normal area was 1.97%, and the correct classification rate was 98.04%.

## 1. Introduction

Touch panels are widely used, among which the capacitive touch panel (CTP) has become the mainstream of the future touch panel market due to its excellent characteristics, including being waterproof, antifouling, scratch resistant, and capable of fast response. In the CTP manufacturing process, defects such as scratches, stains, and foreign objects often occur on the surface of the touch panel due to negligence in the fabrication process. These defects not only affect the appearance of the panel but also reduce the work efficiency of the touch panel and even impair the function of conducting electricity. Therefore, the process inspection of the touch panel cannot be ignored. Nowadays, the defect detection and determination of touch panels are all carried out in the final product stage with considerable labor costs for a comprehensive inspection.

**Figure 1:**
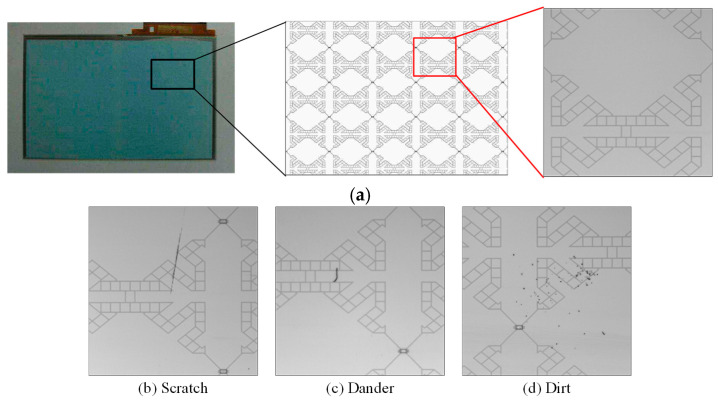
CTP images with directional textured backgrounds. (**a**) A testing sample and a normal image. (**b**–**d**) Defective images with three main defect types.

The surface defects of the touch panel are mostly caused by poor materials, human negligence, or surface damage during the manufacturing and handling process. We can categorize the surface defects of CTPs in the current factories of touch panel manufacturers into six types of defects: touch panel scratches/cracks, dander foreign matter, dirt, watermarks, air bubbles, and edge stretches. Among these, scratches/cracks, dander, and dirt account for the vast majority (more than 80%) of defects in production, and the three types of surface defects are caused by damage to the surface structure of the touch panel. Figure 1 shows a few CTP images with directional textures including (a) a testing 14.7 × 22.7 cm^2^ (thickness 0.78 mm) in size and a normal image and (b)–(d) defective images with three main defect types. The background texture of this sample presents lattice-like lines, the high distribution density is a complex texture, and the lines have about four directions (horizontal, vertical, and two diagonal lines). 

The surface of CTP is distributed with textures, and the textures are all structured textures ordered in repetitive patterns. Panels for different purposes have different background textures according to the type of structure of the touch panel. Table 1 lists six types of background texture patterns of common CTPs. Texture patterns 1 and 2 have more complex background structures (lines with four directions: horizontal, vertical, and two oblique angles) and a higher density of repetitive patterns; texture patterns 3 and 4 have medium background structures (four directions) and moderate density of repetitive patterns; texture patterns 5 and 6 have simpler background structures (two directions: horizontal and vertical) and a lower density of repetitive patterns. Each type of touch panel has a different background texture pattern. This study initially used a more complex background texture sample (texture pattern 1) for flaw detection and discussion.

Due to the strong reflection ability of the touch panel, the surface of the panel is easy to present external objects, which increases the difficulty of detection. The automatic inspection system has the advantages of consistency, accuracy, and cyclic inspection. The development of a well-functioning automatic inspection system can improve quality while reducing manufacturing and rework costs. Since the usage of touch panels is quite large, if an automated inspection system can be used to replace manual inspection, the inspection efficiency and benefits can be greatly improved. Therefore, this study aimed to develop an automated touch panel surface visual inspection system for the most common defects on the surface of CTPs. 

## 2. Literature Review

Automatic detection of appearance defects has become a key issue for manufacturers eager to enhance goods quality and fabrication efficiency [1]. Flaw detection techniques are usually split into spatial and frequency domains [2]. Adamo et al. [3] described a Canny-based edge detection for surface flaw inspection in satin glass. Ng [4] modified the Otsu method for picking optimal thresholds for the distributions of unimodal and bimodal populations and assessed the modified scheme on usual flaw detection tasks. The Otsu method [5] is a better threshold choice scheme for common images pertaining to uniformity and form estimates. In frequency domain technologies, Li and Tsai [6] introduced a wavelet-based discriminant estimate for flaw detection in solar wafer images with uneven backgrounds. Lin [7] implemented a hybrid approach combining the decomposition of discrete cosine transform (DCT) and cumulative sum schemes for flaw inspection in passive components. 

Repeated patterns are common, especially in man-made objects. They provide structural and geometric or semantic clues about the elemental structures of which repeated patterns are composed. The detection of repeated patterns can benefit from many algorithms in computer vision and graphics [8]. The conductive glass for the touch panel is a transparent glass with repeated patterns. The structural textures have regular and homogeneous patterns, and frequency domain conversion is often used to extract texture-related features for defect detection. Lin and Tsai [9] adopted the spectral attributes of the Fourier transform and the multicrisscross filtering to eliminate components with larger energy values to weaken the textured background. Hung and Hsieh [10] employed the attributes of the repeated textural patterns to adaptively revise each textural pattern and then contrast it with the norm pattern to abstract defects. The problem of detecting flaws is complicated by the distinction between the kind of flaws and the textured background of the touch panel images [11]. Chiu and Lin [12] presented a wavelet transform-based method to inspect the flaws in the appearance of touch panels with a variation in the structural background texture. Jian et al. [13] used positioning based on image profiles of the mobile phone screen glass to identify screen flaws. A review of the relevant literature suggests that most related studies inspect and classify the appearance flaws of touch panels [9,10,11,14,15,16].

Directional textures have repeating patterns of fixed orientation and are regularly found in artificial objects, for example, machined components, electronic parts, and fabric textiles. Tsai and Hsieh [17] developed an image restoration technique applying Fourier and Hough transforms to the automated detection of flaws on a variety of artificial surfaces with repeated line patterns. Perng and Chen [18] introduced a method based on nonnegative matrix factorization to automatically inspect for defects on oriented textured surfaces, such as the light-emitting diode panel, the internal thread, and the dioptric pattern of the contact lens. Chen et al. [19] implemented a visual inspection system for inspecting defects in resistive touch panels (RTPs) with periodic spacer textures. Jiang et al. [20] introduced a combined method of nonnegative matrix factorization and tolerance model for defect detection in specific CTP patterns. This algorithm can only be reliably applied in the new-type CTP patterns, which have neither basic primitives nor periodicity. 

In this study, the CTP image employed had a more complicated texture of repeated line patterns. When a panel image with four periodic lines in various directions in the background texture is transformed into the discrete cosine domain, the upper left corner of the cosine spectrum image gathers at least three main bands with high-energy frequencies to radiate out along the three axes. It is hard to accurately identify surface flaws embedded in complex directional textures. Directional textures have regular and analogous patterns, and frequency domain conversion is often used to extract texture-related features for defect detection. The characteristics of the DCT can also show the texture characteristics of the spatial domain image in the discrete cosine spectrum, which has the characteristics of energy concentration, and the image after reconstruction is more similar to the original image. Therefore, we propose a global image restoration approach for small surface defect detection in CTP images using DCT and three-way filtering methods. 

## 3. Research Method

This paper proposes a three-way filtering approach based on a DCT to identify the appearance flaws of CTPs. When a testing image with four various directional line patterns of textured background is converted to a discrete cosine space, three major bands with high-energy frequencies originate from the origin of the cosine spectrum domain and extend along the three axes. With consideration to the bandwidth and angle of the high-energy region in combination with the characteristics of band filtering, threshold filtering, and Gaussian distribution filtering, a three-way double-band Gaussian filter (3W-DBGF) can be devised to sieve the frequencies of the areas of the three main bands and sub-bands. Then, the sieved image is converted back to the spatial space. In the rebuilt image, the analogous line areas in the testing image will have an around even intensity, whereas the flaw area will be distinctly retained. Finally, the rebuilt image is separated using a straightforward binarization scheme, and certain features of the inspected flaws are obtained.

The DCT is specifically designed to concentrate the largest amount of information on the least number of coefficients, which is the toughness of low-frequency coefficients [21]. The DCT transformation process is linear and reversible, so the inverse transformation can correspond to the original data after the forward transformation. The image reconstructed using DCT has fewer errors than does the original image [22]. The main characteristics of the DCT are its adaptive filtering and the phenomenon of fewer blocking artifacts in the spatial/frequency domains [23,24,25]. Since DCT only converts the real part, the operation is faster than is the Fourier transform and does not increase computational complexity [7,26]. Therefore, the proposed DCT-based approach can decrease the effect of various texture angles aroused by distinct piece placements. The influence of various background textures on this approach is less, and more flaws can be detected. 

### 3.1. Two-Dimensional Frequency Transform

The DCT is a method of converting the numerical value of the spatial domain to the frequency domain. In this study, the size of the image to be measured in 2D and the grayscale value of the coordinate (x,y) is represented by f(x,y), and the corresponding 2D DCT can be expressed as follows:

(1) $$D\left(u,v\right)=C\left(u\right)C\left(v\right)\sum_{x=0}^{M-1}\sum_{y=0}^{M-1}f\left(x,y\right)\cos\left[\frac{\left(2x+1\right)u\pi }{2M}\right]\cos\left[\frac{\left(2y+1\right)v\pi }{2M}\right]$$

where x=y=0, 1, 2, 3, …, M−1, and C(u) and C(v) are defined as follows:

$$C\left(u\right)=C\left(v\right)=\{\begin{array}{l} \sqrt{\frac{1}{M}}\;,\;u=0\\ \sqrt{\frac{2}{M}}\;,\;u=v=1,\;2,\;3,\;\ldots ,\;M-1\; \end{array}$$

The discrete cosine spectrum is |*D*(*u*, *v*)| and the power spectrum *P_D_*(*u*, *v*) is defined as follows:

(2) $$P_{D}\left(u,v\right)=\log(1+{\left|D\left(u,v\right)\right|}^{2})$$

**Figure 2:**
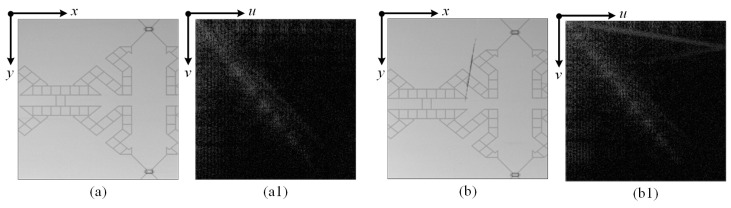
The relevant cosine spectrum images of a normal image (**a**,**a1**) and a defective image (**b**,**b1**).

Figure 2 shows the relevant cosine spectrum images of a normal image (a) and a defective image (b). 

### 3.2. Analyses of Frequency Power Spectrums

The characteristics of DCT can also highlight the repeated lines in the image in the discrete cosine spectrum. In the original image, straight lines with an angle *θ* less than 90 degrees and a complementary angle (180° − *θ*) will be displayed in the direction (*θ* + π/2) of the frequency components in the discrete cosine spectrum. This means that for any straight line with angle *θ* in the image space and the corresponding complementary angle (180° − *θ*) in the discrete cosine spectrum, the two angles will show the energy distribution perpendicular to the angle *θ*. Therefore, the scratches in the vertical direction appear in the image, and in the discrete cosine spectrum, it will be represented by the horizontal energy distribution. 

**Figure 3:**
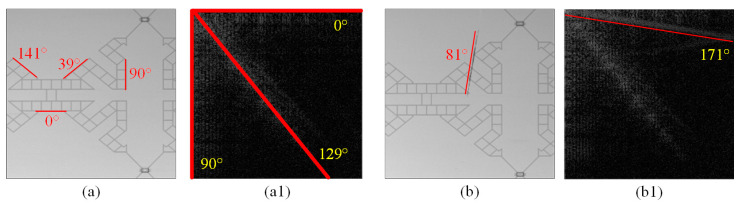
The angular relationship between the surface texture of the testing images (**a**,**b**) and the corresponding discrete cosine spectrum images (**a1**,**b1**).

Figure 3(a1) is the energy spectrum image of the normal image in Figure 3a after DCT transformation. The background texture angles of the normal image in this space are about 4 directions (0°, 39°, 90°, 141°), but the energy spectrum image after DCT transformation has only 3 axes (0°, 90°, 129°), as shown in Figure 3(a1). Since the angle of 39° and the angle of 141° in the original image are complementary, the energy of the two angles (39°, 141°) is distributed in the direction 129° (39° + π/2) of energy on broadband in the discrete cosine energy spectrum image. Two images with the same background texture will have similar energy distributions, but in addition to the three more concentrated energy bands, the defect image obviously has one more energy band, as shown in Figure 3(b1). In the original defect image, a scratch produces a line at another angle, so at least one energy band is added to the discrete cosine energy spectrum, and the energy band on this energy spectrum image will be perpendicular to the original image’s defect direction. 

The energy distribution of the frequency spectrum is also different when different frequency transformations are used, but the spectral characteristics of the energy distribution can all present the background texture characteristics corresponding to the original image. The textured background of the touch panel is concentrated on the main energy bandwidths. Therefore, according to the energy distribution and spectral characteristics of the cosine frequency domain, this study uses specific filters to filter out the range with higher energy to weaken the background texture and retain the flaws. 

### 3.3. Frequency Spectrum Filtering

In order to avoid the occurrence of defects being eliminated, information with higher energy spectrum values is deleted. According to the spectral characteristics and energy distribution of DCT conversion, different filters were respectively designed, and the differences of different filtering area methods are discussed in the subsequent sections. 

#### 3.3.1. Threshold Filtering (TF) Approach

Threshold filtering mainly considers the energy spectrum values of the entire image after conversion in the frequency space and sets an appropriate energy spectrum cutting threshold. After the testing image is forward-transformed into a frequency space using DCT, a cutting threshold $T_{D}$ of an appropriate cosine energy spectrum value is selected in the energy spectrum image for filtering. If the spectrum values of the energy spectrum image satisfy the condition that $P_{D}\left(u,v\right)$ are greater than or equal to the cutting threshold $T_{D}$, all the energy spectrum values are set to $S_{D}$, the spectrum values of the energy spectrum image are then set to 0 (black), the corresponding frequency positions of the image also set the frequency values to 0, and the remaining frequency values maintain the original values. The satisfying condition of threshold filtering is Equation (3), and the frequency values after threshold filtering can be expressed as Equation (4):

(3) $$S_{D}=\left\{P_{D}\left(u,v\right)|P_{D}\left(u,v\right)\geq T_{D}\right\}$$

(4) $$D^{\prime }\left(u,v\right)=\{\begin{array}{cc} 0, & \;\mathrm{if}\;P_{D}\left(u,v\right)\in S_{D}\\ D\left(u,v\right), & \;\mathrm{otherwise}. \end{array}$$

**Figure 4:**
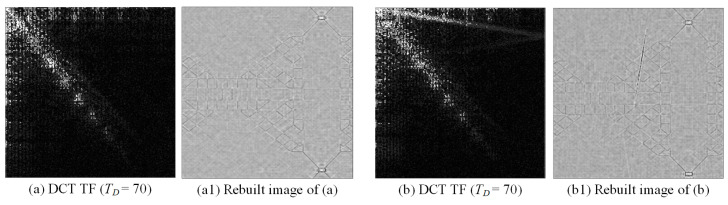
Threshold filtering (TF) method applied to the DCT domain.

Figure 4 shows the process of performing threshold filtering on the discrete cosine energy spectrum image for the normal and defective images, respectively. If the energy spectrum values of the two frequency domains are greater than or equal to the threshold value of 70, then the energy spectrum values of the two frequency domains are deleted, and the corresponding positions of the frequency images are deleted together. The image rebuilding is completed by converting the frequency space back to the spatial space. To highlight the deleted positions in the energy spectrum images, the deleted positions in this study are shown in white (255). 

#### 3.3.2. Band Filtering (BF) Approach

The band filtering method selects the frequency band to be filtered and deletes the frequency values of the entire frequency band, and the threshold filtering method decides whether to delete the frequency value according to whether the size of the energy spectrum value exceeds a preset threshold value. If threshold filtering is used on a defective image, the background texture is removed, but the defect can also be removed. Therefore, in order to avoid such misjudgment of defects, filtering is performed on the regions with high-energy spectral values. To accurately filter high-energy regions, the angles of the main energy bands are measured in the high-energy concentration using discrete cosine spectral images of 10 normal images. In the cosine energy spectrum image, the high energy of the discrete cosine spectrum is concentrated in three axial directions, and the band angles of the three main energy bands in the frequency domain are 0°, 90°, and 129°, respectively.

**Figure 5:**
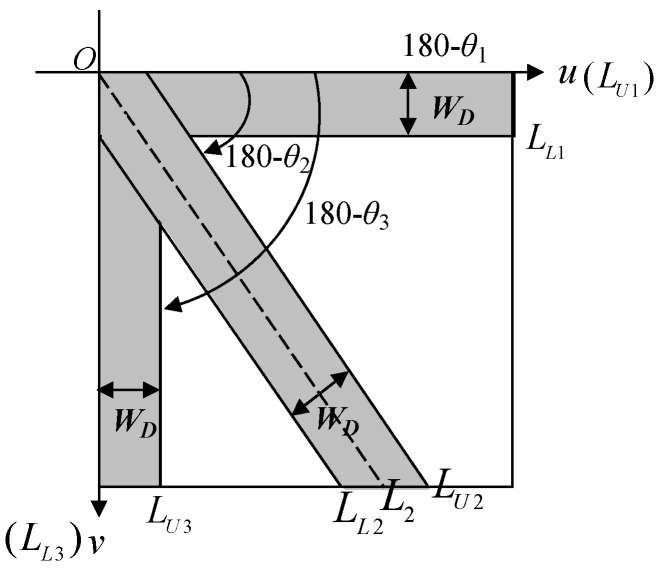
The schematic diagram of the DCT three-way band filtering approach.

There are 3 main energy broadbands in different axes in the DCT frequency space image, so only 3 broadband intervals need to be cut in the DCT frequency domain. $L_{Ui}{}^{*}$ and $L_{Li}{}^{*}$ are the upper and lower boundary ranges of each direction, respectively; (u,v) are the coordinate positions in the frequency space image; and $\theta_{i}$ is the three different broadband angles in the frequency domain, where *i* = 1, 2, 3, are respectively $\theta_{1}=0{}^{\circ}$, $\theta_{2}=51{}^{\circ}$, and $\theta_{3}=90{}^{\circ}$. The schematic diagram is depicted in Figure 5. In the discrete cosine frequency domain image, if a certain broadband range whose direction is $\theta_{1}$, this satisfies Equation (5) as follows:

(5) $$L_{U1}{}^{*}:u\sin\theta_{1}-v\cos\theta_{1}\leq 0;\;\;\;\;\;\;L_{L1}{}^{*}:u\sin\theta_{1}-v\cos\theta_{1}+W_{D}\geq 0.\;$$

If $\theta_{1}=0{}^{\circ}$ is substituted into Equation (5), this simplifies the following equation:

(6) $$L_{U1}{}^{*}:-v\leq 0;\;\;\;\;\;\;\;\;\;\;\;\;\;\;\;\;\;\;\;\;\;\;\;\;\;\;\;\;\;\;L_{L1}{}^{*}:-v+W_{D}\geq 0$$

That is, $L_{U1}{}^{*}\cap L_{L1}{}^{*}=\left\{D\left(u,v\right)|D\left(u,v\right)\in L_{U1}{}^{*}\;and\;D\left(u,v\right)\in L_{L1}{}^{*}\right\}$. Similarly, if a certain broadband range of the center line $L_{2}$ has a direction of $\theta_{2}$, this satisfies Equation (7) as follows:

(7) $$L_{U2}{}^{*}:u\sin\theta_{2}-v\cos\theta_{2}-\frac{W_{D}}{2}\leq 0;\;\;L_{L2}{}^{*}:u\sin\theta_{2}-v\cos\theta_{2}+\frac{W_{D}}{2}\geq 0.\;$$

That is, $L_{U2}{}^{*}\cap L_{L2}{}^{*}=\left\{D\left(u,v\right)|D\left(u,v\right)\in L_{U2}{}^{*}\;and\;D\left(u,v\right)\in L_{L2}{}^{*}\right\}$. Likewise, if a certain broadband range has a direction of $\theta_{3}$, this satisfies Equation (8) as follows:

(8) $$L_{U3}{}^{*}:u\sin\theta_{3}-v\cos\theta_{3}-W_{D}\leq 0;\;\;L_{L3}{}^{*}:u\sin\theta_{3}-v\cos\theta_{3}\geq 0.\;$$

$\theta_{3}=90{}^{\circ}$ can be substituted into Equation (8) to simplify the following equation:

(9) $$L_{U3}{}^{*}:u-W_{D}\leq 0;\;\;\;\;\;\;\;\;\;\;\;\;\;\;\;\;L_{L3}{}^{*}:u\geq 0.\;$$

That is, $L_{U3}{}^{*}\cap L_{L3}{}^{*}=\left\{D\left(u,v\right)|D\left(u,v\right)\in L_{U3}{}^{*}\;and\;D\left(u,v\right)\in L_{L3}{}^{*}\right\}$. This range is the overlapping part of the two regions; that is, the interval range with a width of $W_{D}$ pixels deletes this overlapping range and set its frequency value to D(u,v)=0, and the frequency value of the nonoverlapping range remains the original numerical value. Therefore, the frequency value $D^{\prime }\left(u,v\right)$ after the three-way broadband filtering is Equation (10):

(10) $$D^{\prime }\left(u,v\right)=\{\begin{array}{cc} 0, & \;\mathrm{if}\;D\left(u,v\right)\in L_{Ui}{}^{*}\cap L_{Li}{}^{*}\\ D\left(u,v\right), & \;\mathrm{otherwise}.\; \end{array}$$

The deletion range of this method is three directions $\left(\theta_{1}-\theta_{2}-\theta_{3}\right)$, so this multiband filtering method is referred to as three-way band filtering (3W-BF) in this paper.

**Figure 6:**
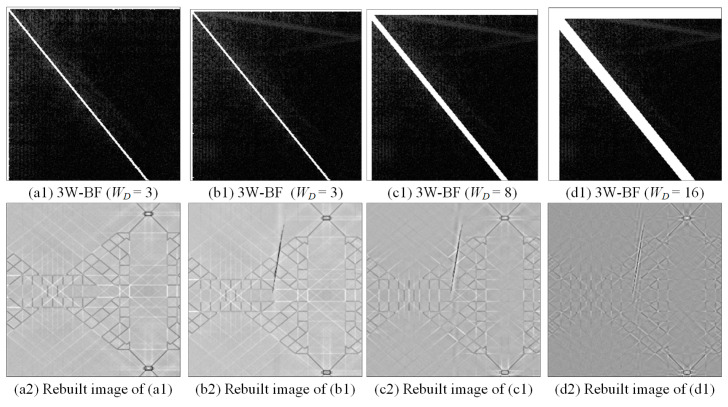
The filtering effects of the DCT three-way band filtering (3W-BF) method with various cutting widths.

Figure 6 shows the performance of three-way band filtering on the discrete cosine energy spectrum images for the normal and the defect images. The bandwidth $W_{D}$ of these frequency domains are 3, 8, and 16 pixels, respectively. The energy spectrum images converted in the frequency domains are in the fixed direction and within the ranges of the bandwidths, so the frequency values in these intervals are deleted in different bandwidth ranges, with the amount of energy deleted being different. If the bandwidth is wider, more energy spectrum values will be deleted. Although the background texture of the rebuilt image has been roughly eliminated, the defects are for the most part removed. Therefore, choosing different bandwidths in different frequency domains has different effects on detection efficiency.

#### 3.3.3. Band-Threshold Filtering (BTF) Approach

Threshold filtering mainly considers the energy spectrum values of the entire image after conversion in the frequency space and sets an appropriate cutting threshold value in the frequency domain. If the energy spectrum values of the energy spectrum image are greater than or equal to the cutting threshold value, the energy spectral values are deleted, but other high-energy spectral components may be unintentionally deleted. The band filtering method involves selecting the location to be filtered and deleting the frequency values in the entire band. Although the main energy distribution extends from the low-frequency band to the high-frequency band, not all the energy spectrum values in the entire band area are high energy components, so it may be that the frequency values that are not high energy are also deleted. In order to avoid deleting other high-energy frequency components and components in non-high-energy regions, this section considers the definitions of the two filtering methods at the same time, with the high-energy components within the band range to be filtered being deleted; thus, as band filtering and threshold filtering methods are combined, this filtering method is called band-threshold filtering (BTF).

**Figure 7:**
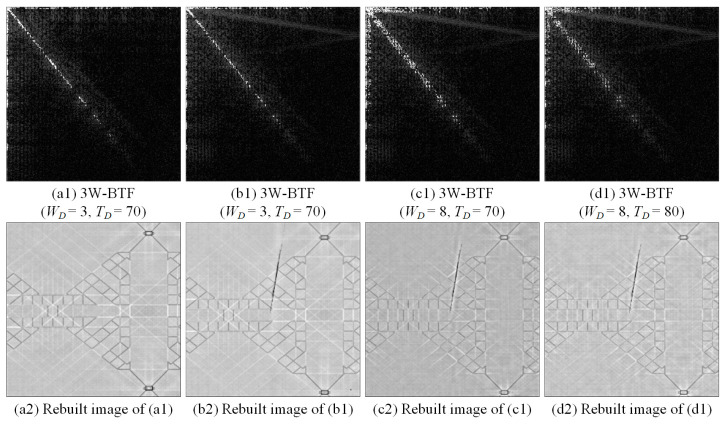
The filtering effects of the DCT three-way band-threshold filtering (3W-BTF) method with various cutting widths and threshold values.

Figure 7 show the process of performing three-way band-threshold filtering on the discrete cosine energy spectrum image for the normal image and the defect image. Under the same cutting threshold in the frequency domain, the deleted energy spectrum values are greater in both the case with the larger bandwidth and the other case with the smaller the frequency domain cutting threshold at the same bandwidth. Although the background texture is weakened to a greater extent, the flaw has been truncated into multiple line segments at the same time. Therefore, the parameter combination of the bandwidth *W* ($W_{F}$,$\;W_{D}$) and the cutting threshold *T* ($T_{F}$,$\;T_{D}$) in different frequency domains is very important, which also affects the detection efficiency. 

#### 3.3.4. Band Gaussian Filtering (BGF) Approach

The band Gaussian filtering (BGF) method assumes that the distribution of energy values of the spectrum image in the bands conforms to the normal distribution. This BGF method needs to have training images, and the parameters of the training images are required as a standard. The average values of the energy spectrum of the training images and the testing images within a certain band must be similar. If they are different, it means that the local energy values of the testing spectrum image have shifted. The same average value is then used to set the control range based on the average value and standard deviation of the energy spectrum of the training images. If the high-energy values in the testing spectrum image exceed this control range, they will be deleted.

**Figure 8:**
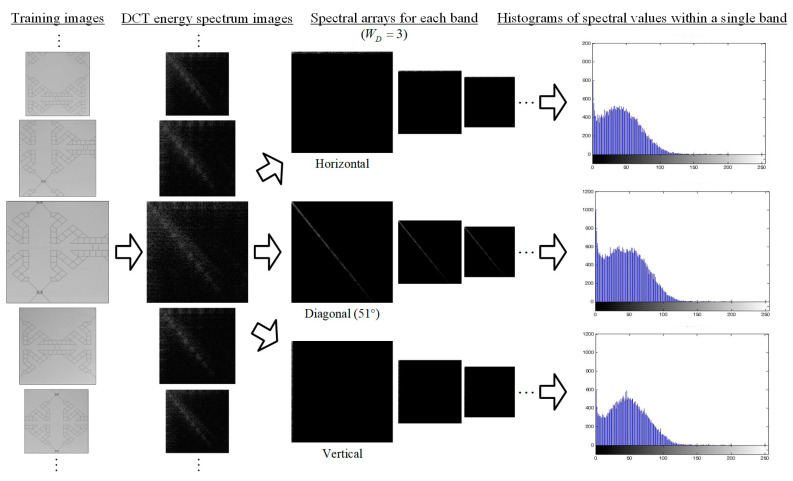
The DCT three-way band Gaussian filtering (3W-BGF) process for training images.

For DCT, the training images are converted into energy spectrum images, and the energy spectrum values in three directions with the main energy are selected to draw three histograms. The histogram distribution in each direction is approximately the normal distribution, as shown in the Figure 8, so this filtering method is called three-way band Gaussian filtering (3W-BGF). The energy value distribution of the corresponding band of the testing image should be the same as that of the training image, so the average and standard deviation of the band energy spectrum of the training image are used as the basis for the control limits of the band energy values of the testing images.

If we calculate the overall average value ($\mu_{Tr\_i}$) and standard deviation ($\sigma_{Tr\_i}$) of the energy spectrum values $P_{D\_i}\left(u,v\right)$ in the horizontal $\left(\theta_{1}=0{}^{\circ}\right)$, diagonal ($\theta_{2}=51{}^{\circ}$), and vertical $\left(\theta_{3}=90{}^{\circ}\right)$ directions of the n training images individually, each direction has a set of average values and the standard deviation, $C_{Tr}$ is the number of pixels in the band range of a certain direction of the energy spectrum image, and the total mean and standard deviation are calculated as Equations (11) and (12) as follows:

(11) $$\mu_{Tr\_i}=\frac{\sum_{1}^{n}P_{D\_i}^{n}\left(u,v\right)}{n\cdot C_{Tr\_i}}$$

(12) $$\sigma_{Tr\_i}=\sqrt{\frac{\sum_{1}^{n}{\left[P_{D\_i}^{n}\left(u,v\right)-\mu_{Tr\_i}\right]}^{2}}{\left(n\cdot C_{Tr\_i}-1\right)}}$$

We can set the control limit interval of each direction to be $\left(\mu_{Tr_{i}}+\lambda \;\sigma_{Tr\_i}\right)$, with λ being the energy threshold coefficient in the band. 

If the average value of the energy spectrum of the testing image is not equal to the average value of the total energy spectrum of the training images, it means that the overall distribution has shifted, so the offset ($\delta_{i}=(\mu_{Tr\_i}-\mu_{Te\_i}$) must be calculated. Then, the energy spectrum value $P_{D\_i}\left(u,v\right)$ of the testing energy spectrum image is moved (increased or decreased) to the same average energy spectrum value as the training energy spectrum images. The energy spectrum values of increments $P_{D\_i}^{\#}\left(u,v\right)$ in 3 directions are calculated in Equation (13) as follows:

(13) $$P_{D_{i}}^{\#}\left(u,v\right)=\{\begin{array}{c} P_{D_{i}}\left(u,v\right),{\;\mathrm{if}\;P}_{D_{i}}\left(u,v\right)\in L_{U_{i}}{}^{*}\cap L_{L_{i}}{}^{*}{\;\mathrm{and}\;\mu }_{Tr_{i}}=\mu_{Te_{i}}\\ P_{D_{i}}\left(u,v\right)+\delta_{i},{\;\mathrm{if}\;P}_{D_{i}}\left(u,v\right)\in L_{U_{i}}{}^{*}\cap L_{L_{i}}{}^{*}{\;\mathrm{and}\;\mu }_{Tr_{i}}\neq \mu_{Te_{i}} \end{array},\;\;\;$$

where $L_{U_{i}}{}^{*}$ and $L_{L_{i}}{}^{*}\;$ are the range of Equations (5) and (9) in the discrete cosine spectrum within a certain bandwidth. 

If the energy spectrum value of the testing image is greater than the control limit $\left(\mu_{Tr_{i}}+\lambda \;\sigma_{Tr_{i}}\right)$ set by the training images, the frequency value of the corresponding location of the frequency domain image is set to 0 (delete). (The position to be deleted can be clearly known from the energy spectrum image, but the real deletion position is in the frequency domain image.) The filtered frequency value $D^{\prime }\left(u,v\right)$ is as follows:

(14) $$D^{\prime }\left(u,v\right)=\{\begin{array}{c} 0,\begin{array}{c} \mathrm{if}\;D\left(u,v\right)\in L_{U_{i}}{}^{*}\cap L_{L_{i}}{}^{*}\\ \mathrm{and}\;P_{D\_i}^{\#}\left(u,v\right)>\left(\mu_{Tr\_i}+\lambda \;\sigma_{Tr\_i}\right) \end{array}\\ D\left(u,v\right),\;\mathrm{otherwise}\; \end{array}$$

**Figure 9:**
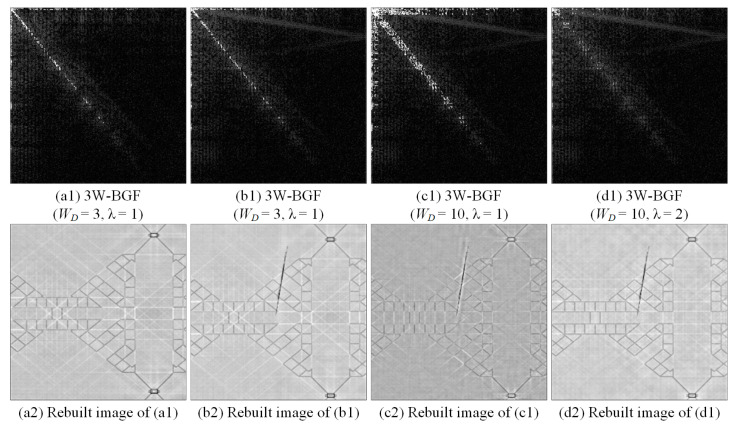
The filtering effects of the DCT three-way band-Gaussian filtering (3W-BGF) method with various cutting widths and energy threshold values.

Figure 9 shows the filtering effects of the DCT three-way band-Gaussian filtering (3W-BGF) method with various cutting widths and energy threshold values. We discuss the influence of the same band energy threshold coefficient (λ) on the detection efficiency of different bandwidths $\left(W_{D}\right)$ and the influence of the same bandwidth on the detection efficiency of different band energy threshold coefficients. At the same band energy threshold coefficient, when the bandwidth is larger, more energy spectrum values are deleted. In the same bandwidth, the smaller the band energy threshold coefficient is, the more energy spectrum values are deleted. Therefore, the parameter combination of the bandwidth and the band energy threshold coefficient is very important and has a degree of impact on the detection effect. 

#### 3.3.5. Double-Band Gaussian Filtering (DBGF) Approach

**Figure 10:**
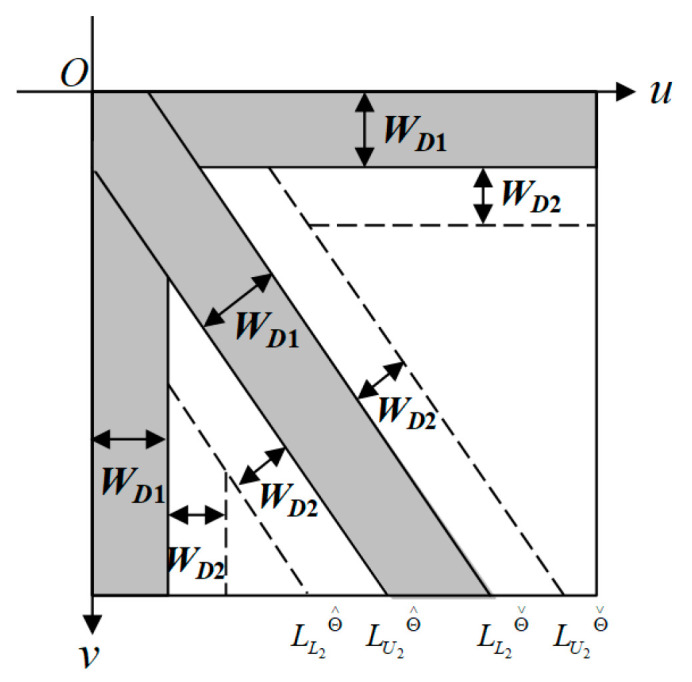
Schematic diagram of the widths of the three-direction main bands and sub-bands of the 3W-DBGF filter.

The three-way band Gaussian filtering is further extended to three-way double-band Gaussian filtering (3W-DBGF). The double-band Gaussian filter not only considers the three band ranges with concentrated energy, but also discusses the increased band areas on both sides of the main band range. Therefore, the main bands in the horizontal and vertical directions are only on one side and one band interval is added to each, and two band regions are added on both sides of the main band in the oblique direction, so a total of 7 bandwidths need to be considered. The 3W-DBGF filter considers the main band and sub-bandwidths and the Gaussian filtering method at the same time. Figure 10 shows the schematic diagram of the widths of the three-direction main bands and sub-bands of the 3W-DBGF filter.

The average value of the energy spectrum in the sub-bandwidth is shifted to be $P_{D\_i}^{\#}\left(u,v\right)$, and the control limit set by the energy spectrum value of the sub-broad bandwidth of the training image is $\left(\mu_{Tr\_i}+\lambda_{2}\sigma_{Tr\_i}\right)$. If the energy spectrum value in the sub-bandwidth is greater than the threshold of this control limit, then its frequency value is set to D(u,v)=0, and the frequency value that does not meet this range will retain the original values.

**Figure 11:**
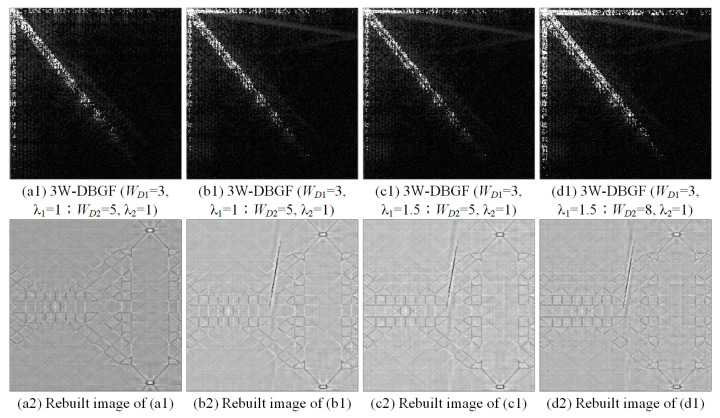
The filtering effects of the DCT-based 3W-DBGF method with various main band and sub-bandwidths and energy threshold values.

Figure 11 shows the filtering effects of the DCT-based 3W-DBGF method with various cutting widths and energy threshold values. We discuss the influence of the same band energy threshold coefficients ($\lambda_{1}$,$\;\lambda_{2}$) on the detection efficiency of different main band and sub-bandwidths ($W_{D1}$,$\;W_{D2}$) and the influence of the same bandwidth on the detection efficiency of the different band energy threshold coefficients. At the same band energy threshold coefficients, when the bandwidth is larger, more energy spectrum values are deleted. In the same bandwidths, the smaller the band energy threshold coefficients are, the more energy spectrum values are deleted. Although the structure of the background texture is destroyed to a large extent, the overall gray value of the image is reduced, and the entire image is darker. This does not significantly help blemish enhancement. Therefore, the parameter combination of the main band and sub-bandwidths and the band energy threshold coefficients is very important and has a relative impact on the detection effect. 

### 3.4. Image Rebuild and Defect Segmentation

After inverse DCT transformation on all pixels (*u*, *v*) in the cosine space image is conducted, the filtered image is rebuilt to the spatial space as follows:

(15) $$f^{\prime }\left(x,y\right)=\sum_{u=0}^{M-1}\sum_{v=0}^{M-1}C\left(u\right)C\left(v\right)D\left(u,v\right)\cos\left[\frac{\left(2x+1\right)u\pi }{2M}\right]\cos\left[\frac{\left(2y+1\right)v\pi }{2M}\right]\;$$

for *x*, *y* = 0, 1, 2, …, *M* − 1. The rebuilt image *f*^′^ (*x*, *y*) will be roughly a uniform intensity image if a flawless image is evaluated. 

After the filtered testing image is rebuilt to the spatial domain, the interval limit is estimated as the binarization threshold $\left(T_{R}^{f}\right)$ of the rebuilt image to separate the defect and the background. It can be expressed as follows:

(16) $$T_{R}^{f}=\mu_{f^{\prime }}+k_{f^{\prime }}\sigma_{f^{\prime }}$$

where $k_{f^{\prime }}$ is a decision parameter, and $\mu_{f^{\prime }}$ and $\sigma_{f^{\prime }}$ are the grayscale mean and standard deviation of the reconstructed image of size M × M, respectively. After binarization with the threshold $T_{R}^{f}$, the binarized image $B_{f}\left(x,y\right)$ can be obtained as follows:

(17) $$B_{f}\left(x,y\right)=\{\begin{array}{c} 255\;,\;\mathrm{if}\;f^{\prime }\left(x,y\right)\leq T_{R}^{f}\\ 0\;,\;\mathrm{otherwise}. \end{array}$$

If the grayscale value of the image is less than or equal to the threshold value, it is set to 255 (white); that is, it is judged as a normal area (background texture); otherwise, it is set to 0 (black); that is, it is judged as a defective area. 

## 4. Experiments and Results

To verify the feasibility of the proposed approach, systematic development and verification experiments were carried out for the proposed methods to determine whether the methods can achieve the expected detection effect of touch panel surface defects. This study established a visual inspection system for touch screen surface defect detection, including software and hardware for image acquisition and inspection software developed according to the research methods. In our experiments, the image size was 256 × 256 pixels, and the number of samples was 30 flawless images for training and 148 images for testing, for which there were 112 flawed images and 36 flawless images, respectively.

For the performance metrics of flaw inspection, we used the false-discovery rate for normal regions (α), the defect detection rate for defective regions (1 − β), and the correct classification rate for all regions (CR). The false-discovery rate is the normal region divided by the area of the regular districts detected as flaws by the areas of the real normal districts. The defect detection rate of the real defect regions is the areas of the inspected true defects divided by the area of the overall real defects. The correct classification rate is the correctly inspected districts divided by the total district of an image. In this study, (μ + k σ) was used to set the threshold value to explore the influence of different parameter k values on α and (1 − β) and to draw the receiver operating characteristic (ROC) curve with α as a horizontal axis and (1 − β) as a vertical axis to decide the value of the parameter k. Therefore, the point k closest to the upper left corner of the ROC curve is the case where the value of α is the lowest and the value of (1 − β) is the highest, which was the test result expected by this study. The position of the ROC curve corresponding to the detection test with increasing discriminative ability gradually approaches the upper left corner of the ROC space. The curve closest to the upper left and containing the other curves has a greater discriminate capacity than does the other curves. After better parameters for each filtering method were determined individually, filtering methods with different parameter combinations were selected by comparing the area under the ROC curve (AUC). In industrial practice, a more than 90% detection rate (1 − β) and a less than 5% false-discovery rate α are good rules of thumb for the performance evaluation of a vision system [9].

### 4.1. Performance Evaluation of the Explicit Filtering Methods with Various Parameter Settings

#### 4.1.1. Bandwidths and Frequency Thresholds of Filtering in BTF Approach

**Figure 12:**
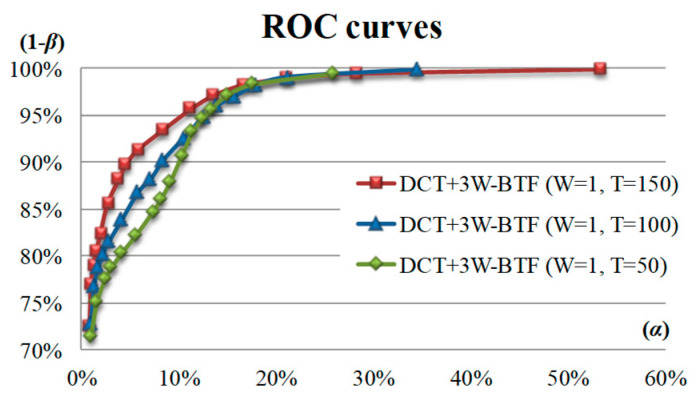
ROC curves of the thresholds of 50, 100, and 150 using the DCT-based 3W-BTF method with a bandwidth of 1.

In the DCT-based 3W-BTF method, the parameters of the band threshold filtering method are the bandwidth ($W_{D}$) and the frequency threshold ($T_{D}$), and the influence of different two-parameter combinations on the detection efficiency is discussed. Figure 12 shows the detection effect ROC curves of the thresholds of 50, 100, and 150 using the DCT-based 3W-BTF method with a bandwidth of 1. The detection result shows that under a fixed α value and a fixed bandwidth of 1 pixel, the ROC curve with a threshold value of 150 is higher than the two ROC curves with threshold values of 50 and 100, thus indicating a larger detection rate (1 − β). Therefore, under the same bandwidth, the larger the threshold is, the less the filtered values and the better the detection result. Therefore, the detection effect is better when the bandwidth ($W_{D}$) is 1 pixel and the frequency filtering threshold ($T_{D}$) is 150.

#### 4.1.2. Bandwidths and Energy Threshold Coefficients of Filtering in DBGF Approach

**Figure 13:**
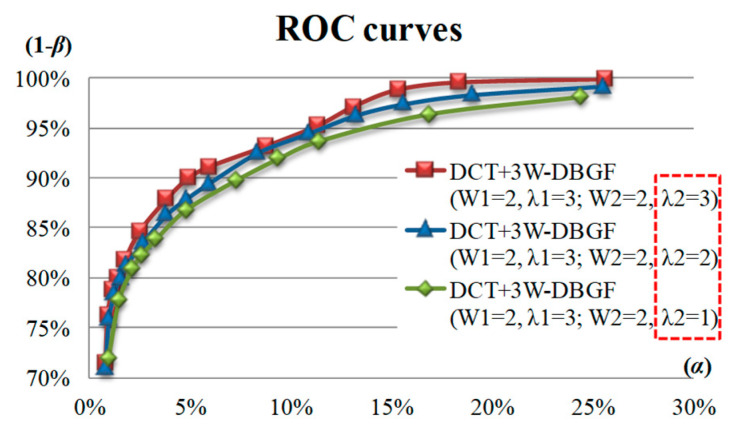
ROC curves of the $\lambda_{2}$ values of 1, 2, and 3 using the DCT-based 3W-DBGF method with three preset fixed parameters.

In the DCT-based 3W-DBGF method, the parameters of the double band-Gaussian threshold filtering method are the two sub-bandwidths ($W_{D1},\;W_{D2}$) and the sub-band energy threshold coefficients $\left(\lambda_{1},\;\lambda_{2}\right)$, and the influence of different two-parameter combinations on the detection efficiency are discussed. Figure 13 shows the detection effect ROC curves of the $\lambda_{2}$ values of 1, 2, and 3 using DCT-based 3W-DBGF method with three preset fixed parameters, ($W_{D1}$, $W_{D2}$) = (2, 2) and ($\lambda_{1}$ = 3). The detection result shows that under a fixed α value and three fixed parameters, the ROC curve with a sub-band threshold coefficient of 3 is higher than the two ROC curves with sub-band threshold coefficients of 2 and 1, that is, there is a larger detection rate (1 − β). Therefore, the detection effect is better when the parameter combinations of sub-bandwidths and the sub-band energy threshold coefficients are ($W_{D1}$ = 2, $\lambda_{1}$ = 3; $W_{D2}$ = 2, $\lambda_{2}$ = 3).

### 4.2. Comparisons of the Different Band Filtering Methods

According to the small sample experiment, for each frequency domain transformation with different band filtering methods, after the parameter combinations with better detection effects are set, the areas under the ROC curves of the 8 filter combinations are calculated quantitatively. According to the size of the area, the frequency domain conversion is selected individually with a certain broadband filtering method. According to the size of the area, a specific band filtering method is selected with the corresponding frequency transformation. Table 2 presents the relative areas under the ROC curves for the 4 filter combinations for the same fixed range of α and (1 − β). Since the performance indicators of the testing results are concentrated in a certain range, the range of α as 0–60% and the range of (1 − β) as 70–100% are used to predict the trend line of the ROC curve, and the trend line uses the logarithmic function model. The results show that the area under the ROC curve of the DCT-based 3W-DBGF method has the highest area under the ROC curve (97.94%) with the parameter settings $W_{D1}$ = 2, $\lambda_{1}$ = 3 and $W_{D2}$ = 2, $\lambda_{2}$ = 3. Therefore, it is better to use DCT with 3W-DBGF for the detection results of α and (1 − β). A large sample experimental analysis was conducted for the filtering combination method. 

### 4.3. Large-Sample Experiments

**Figure 14:**
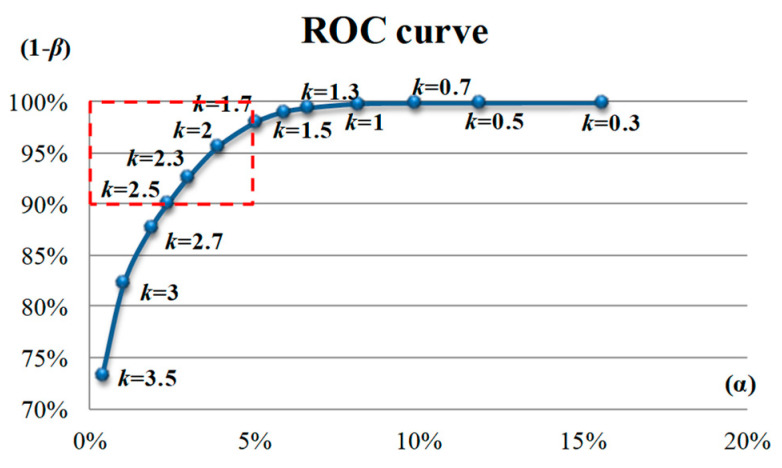
The ROC curve of various binarization threshold *k* values obtained using the DCT-based 3W-DBGF method in large-sample experiments.

**Figure 15:**
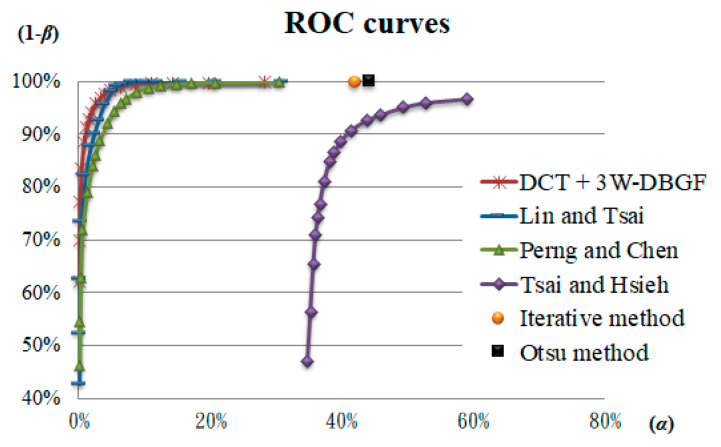
ROC curves of detection performance of the different detection methods.

In the large sample experiments, 148 CTPs with a size of 256 × 256 pixels were used as testing images, of which 112 were defective images and 36 were nondefective images. The parameters of the DCT based 3W-DBGF method were set to ($W_{D1}$ = 2, $\lambda_{1}$ = 3; $W_{D2}$ = 2, $\lambda_{2}$ = 3) to perform defect detection in the large-sample experiments. Figure 14 presents the ROC curve of the detection effect of different binarization thresholds (*k*) in the large-sample experiment. The *k* value closest to the upper left corner is a better *k* value. The *k* values in the box in Figure 15 from 2 to 2.5 all meet this standard, and the closest to the upper left corner is *k* = 2.3. 

### 4.4. Comparison of Performance Evaluation Indexes of Different Detection Methods

The frequency domain methods were used mainly in this study, so the spatial domain methods and other related methods are discussed to evaluate the detection performance of touch panel defect detection. Common spatial domain methods are Iterative [21] and Otsu [5], which directly use the calculated binarization threshold to process the original spatial image. The frequency domain methods can be compared with those of Tsai and Heish [17], who proposed a band filtering (BF) method using Fourier transform with Hough transform; Perng and Chen [18], who proposed a threshold filtering (TF) method using DCT with Rosin unimodal threshold; and Lin and Tsai [9], who proposed a multicrisscross band filtering (MC-BF) method using Fourier transform. All were applied to the testing objects with the directional background patterns and were applied for defect detection. In this study, 148 testing images were used to detect surface defects in the touch panels in the spatial and frequency domains, and the evaluations of the detection effect and efficiency were compared. We drew the corresponding ROC curves according to the *k* values of different parameters and selected the inspection standards of this study. Figure 15 shows the ROC curves of the frequency domain methods and the related research methods. The results reveal that although the spatial domain methods have higher defect detection rates, the false-positive rates of the normal areas are relatively high. Therefore, the spatial domain methods are less suitable for detecting defects in the testing samples for this study. The ROC curves of the methods proposed by Tsai and Hsieh [17], Perng and Chen [18], and Lin and Tsai [9] are lower than the ROC curves of our proposed method, with α being significantly higher than the detection results of this study and (1 − β) being lower. Therefore, the proposed method has better detection outcomes, as shown in Table 3. The proposed DCT-based 3W-DBGF method has 94.21% for (1 − β), 1.97% for α, and 98.04% for CR. The detection time of the DCT based filtering method takes 1.62 s, which is relatively time-consuming compared to other methods, mainly because the main bands and sub-bands in seven band ranges are considered. 

**Figure 16:**
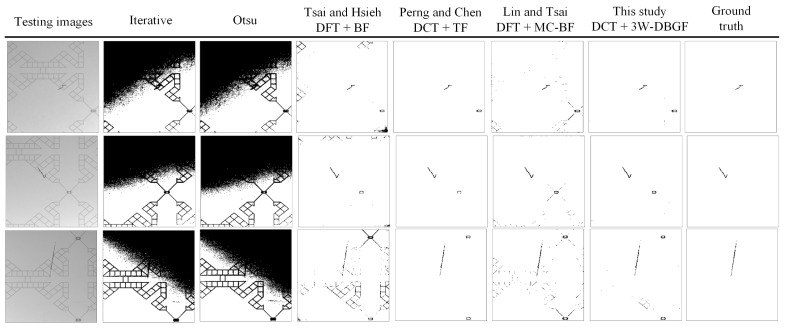
Partial resulting images of different detection methods.

Figure 16 shows images of the results of this study using spatial domain and frequency domain methods with comparison to images tested by other professionals. The results show that the spatial domain method is less effective for the inspection of surface defects of the touch panel and cannot correctly distinguish a flaw from a background. the Tsai and Hsieh’s [17] method detects the angle of the high-energy band and removes the frequency values within its band to highlight flaws; however, the precision of the angle selected with the Hough transform is very important, and other angles can cause errors in filtering the band range, so the probability of the background of some images being misjudged as defects is quite high. Although Perng and Chen [18] used DCT with the Rosin single-peak threshold method, mainly to remove high-energy components, the results show that the location of the defect can be clearly judged, but the false-positive rate of the normal area is slightly higher. After various defect detection methods were performed, the results were statistically evaluated and analyzed, showing that the detection indicators of the proposed method are better than those of other methods. 

### 4.5. Robustness Testing of Flaw Detections for Various Cutting Angles and Background Textures with the Proposed Methods

#### 4.5.1. Performance of Using Different Band Angle Filters on Defect Detection

**Figure 17:**
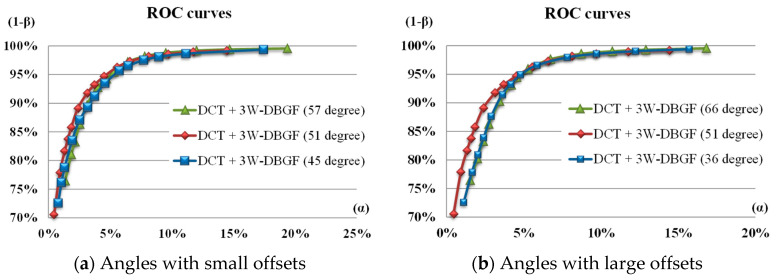
ROC curves of the detection benefit using the DCT-based 3W-DBGF method for (**a**) small offsets (±6°) and (**b**) large offsets (±15°) in band angles.

**Figure 18:**
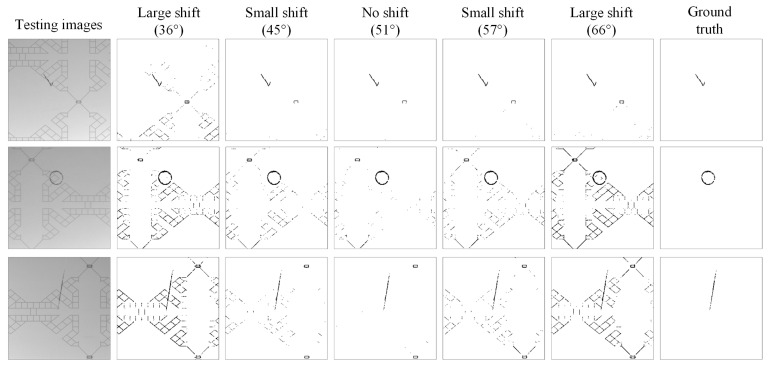
Partial detection results of using the DCT-based 3W-DBGF method for various levels of offsets in band angles.

In the DCT energy spectrum image, the high-energy values are relatively concentrated in the band locations of three angles (0°–51°–90°). These three areas need accurate filtering for defect detection. We investigated whether the change of the oblique angle has an impact on the detection efficiency. The oblique angle was changed from 51 degrees to a small offset of 6 degrees and a large offset of 15 degrees, while the bandwidth angles in the horizontal and vertical directions remained unchanged at 0 degrees and 90 degrees. Figure 17 shows the ROC curves of detection benefit using the DCT-based 3W-DBGF method for small offsets (±6°) and large offsets (±15°) in band angles. The DCT-based approach showed no significant difference in the detection results when the band angles had small offsets; however, when the band angles had large offsets, this had a significant impact on the detection results, but the detection results of accurate filtering were still better. Figure 18 shows the partial detection results of using the DCT-based 3W-DBGF method for various levels of offsets in band angles. 

#### 4.5.2. Detection of CTP Images with Different Background Textures

**Figure 19:**
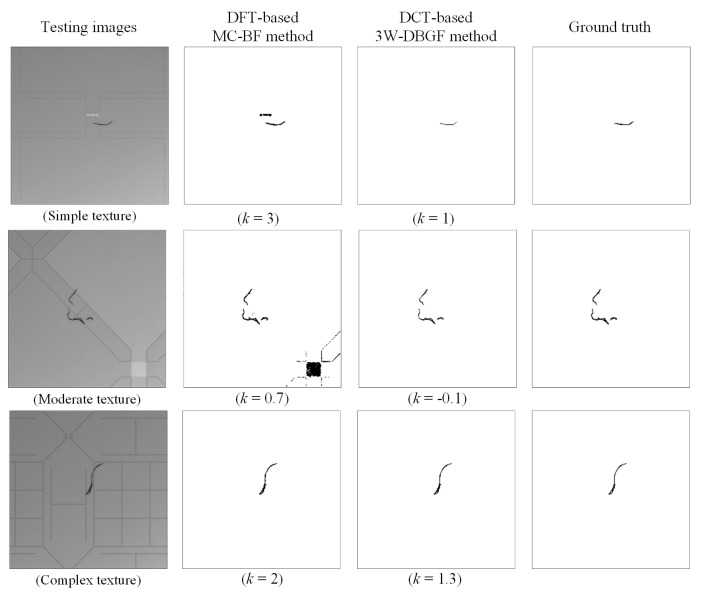
Partial detection results of CTPs with three different complexity levels of background textures obtained by the proposed methods.

In order to expand the detection of objects in this study, the surface defects of touch panels with various background textures were detected, and their detection performance was compared. In Table 1, three common types of CTPs with background textures are presented, namely background textures 2, 3, and 5, while the previous experimental samples in this study are background texture 1 with complex textures, and the surface lines are all structural and directional textures. In this study, the two frequency-domain-based combined filtering methods proposed previously were applied to the three different background textures for defect detection. The parameter settings of the different texture backgrounds were also different, as shown in Table 4, which also summarizes the performance assessment indicators of defect inspection results of CTPs with three distinct complexity levels of background textures using the DFT-based MC-BGF and DCT-based 3W-DBGF methods. For defect detection of three types of background textures with different complexities, the proposed DCT based 3W-DBGF method still showed good detection benefits for the structural distribution of different background textures. Figure 19 shows the partial detection results of CTPs with three different complexity levels of background textures obtained by the proposed methods.

## 5. Conclusions

This study proposes a frequency space filtering approach to detect surface defects of CTPs with directional textures. This method can mainly reduce the influence of defects on spatial domain images by background texture interference, thereby avoiding the false detection of defects. The DCT is used to first convert the image from the spatial space to a spectral image in the frequency domain. A three-way double-band Gaussian filter (3W-DBGF) approach with threshold filtering, double-band filtering, and Gaussian characteristics is proposed. The method first calculates the average of energy spectrum values within the main and sub bands in the testing image and moves it to the position of the total energy spectrum average within the same bands as the normal image in the training set. Then, the interval estimation scheme is applied to set the broadband energy threshold coefficient to delete high energy values in the band ranges. The remaining spectral values are finally inverted back to the spatial space, and this rebuilt filtered image has the effect of reducing background texture and enhancing defects. Experimental outcomes show that the proposed approach achieved better inspection results in detecting CTP surface defects with oriented background textures, with 94.21% (1 − β), 1.97% (α), and 98.04% (CR). In the sensitivity analysis, when the band angle was small offset or the variation of image brightness was medium, this had little effect on the detection results of the proposed method. In the detection of different background textures, the use of the DCT filtering combination method produced good detection results for texture types of differing complexity.

## Figures and Tables

**Table 1:** Six common types of surface background texture patterns of CTPs.

Types	Pattern 1	Pattern 2	Pattern 3	Pattern 4	Pattern 5	Pattern 6
Texture complexity	Complex texture	Complex texture	Moderate texture	Moderate texture	Simple texture	Simple texture
Texture figure	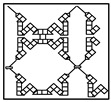	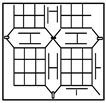	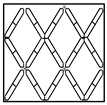	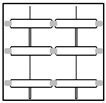	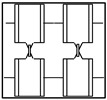	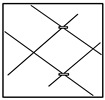
Texture image	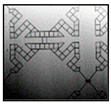	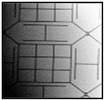	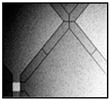	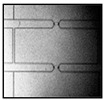	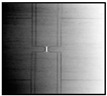	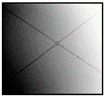

**Table 2:** Areas under the ROC curves and parameter settings for the DCT domain and various band filtering methods.

Band Filtering Methods	(*α*)%: 0~60% and (1 − *β*)%: 70~100%			
	3W-BF	3W-BTF	3W-BGF	3W-DBGF
Parameter settings	($W_{D}\;$= 1)	($W_{D}\;$= 1, $T_{D}\;$= 150)	($W_{D}\;$= 1, λ = 3)	($W_{D1}\;$= 2, $\lambda_{1}\;$= 3; $W_{D2}\;$= 2, $\lambda_{2}\;$= 3)
AUC (%)	94.93	97.46	97.57	97.94

**Table 3:** Performance evaluation comparison table of the different methods for CTP defect detection.

Indicator	Spatial Domain		Frequency Domain			
	Iterative [21]	Otsu [5]	Tsai and Hsieh [17]	Perng and Chen [18]	Lin and Tsai [9]	Proposed Method
			DFT + BF	DCT + TF	DFT + MC-BF	DCT + 3W-DBGF
*k*	--	--	2.3	2.3	2.3	2.3
1 − *β* (%)	99.89	99.94	76.75	88.78	92.72	94.21
*α* (%)	41.84	44.16	36.68	3.23	2.98	1.97
CR (%)	58.26	55.95	63.38	96.75	97.01	98.04
Time (s)	0.0078	0.0047	1.03	0.26	2.96	1.62
Filtering parameter	--	--	*W* = 1	*T* = 100	$W_{F}\;$= 1, λ = 0.5	($W_{D1}\;$= 2,$\;\lambda_{1}\;$= 3; $W_{D2}\;$= 2,$\;\lambda_{2}\;$= 3)

**Table 4:** CTPs with three different complexity levels of background textures, parameter settings, and performance indicators of defect detection results obtained using the DCT-based 3W-DBGF method.

	Complex Texture (Background Texture-2)	Moderate Texture (Background Texture-3)	Simple Texture (Background Texture-5)
Sample images (Normal samples)	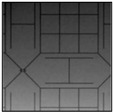	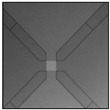	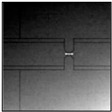
Parameter settings	($W_{D1}\;$= 2, $\lambda_{1\;}$= 3; $W_{D2}\;$= 2, $\lambda_{2}\;$= 3)	($W_{D1}\;$= 2, $\lambda_{1}\;$= 1; $W_{D2}\;$= 2, $\lambda_{2\;}$= 1)	($W_{D1}\;$= 1, $\lambda_{1}\;$= 0.5; $W_{D2}\;$= 1, $\lambda_{2}\;$= 0.5)
*k*	1.3	−0.1	1
1 − *β* (%)	93.11	92.06	92.66
*α* (%)	0.53	3.02	0.31
CR (%)	99.44	87.73	99.68

## Data Availability

Not applicable.
